# Effects of Continuous and Accumulated Exercise on Endothelial Function in Rat Aorta

**DOI:** 10.5935/abc.20170036

**Published:** 2017-04

**Authors:** Juliana Edwiges Martinez, Elane de Fátima Taipeiro, Agnaldo Bruno Chies

**Affiliations:** Faculdade de Medicina de Marília, Marília, SP - Brazil

**Keywords:** Rats, Exercise, Physical Fitness, Endothelium, Acetylcholine, Norepinephrine, Weight Loss

## Abstract

**Background:**

The practice of exercise in short bouts repeated throughout the day may be an alternative strategy to lift people out of physical inactivity.

**Objective:**

to evaluate if accumulated exercise, as occurs in continuous exercise training, improve endothelial function in rat aorta.

**Methods:**

Wistar male rats were divided into three groups: continuous exercise (CEx, 1 hour on the treadmill) or accumulated exercise (AEx, 4 bouts of 15 minutes / day) for 5 days/week for 8 weeks, or sedentary (SED). During the training period, body weight gain and increase in exercise performance were recorded. On sacrifice day, aorta was dissected into rings (3-5 mm) and mounted on the organ bath.

**Results:**

Fitness was significantly greater in CEx and AEx rats as compared with SED animals. In addition, compared with the SED group, CEx animals had a lower body mass gain, and the aorta obtained from these animals had reduced contractile response to norepinephrine and greater acetylcholine-induced relaxation. These results were not observed in ACEx animals.

**Conclusions:**

Both CEx and AEx improved fitness, but only CEx led to reduced body weight gain and improved endothelial function.

## Introduction

Exercise has been considered an important instrument for the promotion of health and prevention of cardiovascular diseases. It is defined as any "physical activity that is planned, structured, and repetitive and [that] has as a final or intermediate objective the improvement or maintenance of physical fitness".^1,2^ The pattern of regular exercise that brings better health benefits is still debated in the literature. Normally, it is recommended exercise of moderate intensity, at least 3 days a week.^3^ Alternatively, exercise may be performed by bouts of at least 10 minutes of high intensity exercise interspersed with intervals of recovery, i.e., periods of mild exercises or simply rest.^4,5^ On the other hand, current recommendations also suggest that short sessions of moderate-intensity physical activity accumulated throughout the day to attain a daily goal of 30 min of exercise - named accumulated exercise^2^ - may be employed to improve the health or as adjuvant treatment of cardiovascular diseases.^6^ Indeed, the practice of exercise in accumulated sessions can be an alternative to lift people out of physical inactivity.^7^

Health benefits of the accumulated exercise have already been demonstrated: elevation of high-density lipoprotein levels,^8,9^ reduction of postprandial triglycerides,^10^ blood pressure levels,^11,12^ skinfold thickness and waist circumference,^6^ and improvement of fitness^13^ and mood state.^6^ However, there is no evidence of the influence of accumulated exercise on endothelial function.

The benefits of exercise on endothelial function occur mostly by the increment of shear stress on endothelial surface, thereby stimulating the expression of endothelial nitric oxide synthase (eNOS), cyclooxygenase-2 (COX-2) and superoxide dismutase-1 (SOD- 1).^14-17^However, it has been demonstrated in endothelial cell cultures that the exposure time to the shear stress influences the expression of these enzymes.^15,18^ Particularly in relation to eNOS, it was demonstrated that the shear stress exposure time influences its degree of phosphorylation, thus regulating its activity.^19^ Thus, it is reasonable to infer that the exposure to different exercise times may have different effects on the expression of endothelial enzymes. Thus, the aim of the present study was to verify whether training by accumulated exercise improves endothelial function in rat aorta such as it occurs in consequence of training by continuous exercise.

## Methods

### Animals

Thirty three male Wistar rats weighing 300-400 g were housed in plastic cages (50 x 40 x 20 cm), 5 animals per cage, with food and water "ad libitum". The sample size (n) was established on the basis of studies that evaluated the effects of continuous exercise on endothelial function.^16,20^ Notably, these studies were the basis for the present investigation that investigate the cardiovascular effects of accumulated exercise. During the exercise protocol, the animals were maintained in the training room under a 12 h light-dark cycle beginning at 7:00 h, at room temperature (25°C). This study was approved by the Ethics Committee on Animal Use of Marilia School of Medicine (protocol n° 627/13).

### Exercise protocol

Rats were initially trained to walk on a treadmill (Movement Technology LX 170) then submitted to daily sessions of 10 minutes, from 0.3 up to 0.5 km/h, without slope, for 2 weeks. At the end of this period, the animals were submitted to the treadmill running test, consisted of graded treadmill exercise at increments of 0.3 km/h every 3 minutes, starting at 0.3 km/h and increased up to the maximal intensity attained for each rat. Based on the results in this test, the animals were randomly assigned to one of the following groups: sedentary (SED), trained by continuous exercise (CEx) or trained by accumulated exercise (AEx), with a similar mean maximal exercise capacity in each group. Subsequently, the animals of the CEx group were exposed to this exercise 5 days per week, 1 hour per day (starting at 09:00am) for 8 weeks. The exercise intensity was increased progressively by a combination of time and velocity, to a maximum of 2 hours per day at a velocity correspondent to 60% of maximal exercise capacity, which was attained by the third week. In parallel, the animals belonging to the AEx group were submitted to 4 short exercise sessions (15 minutes, at similar speed to the CEx group), regularly distributed throughout the day (starting at 07:30am, 10:25am, 01:05pm and 03:45pm), 5 days per week, for 8 weeks. Rats allocated to the SED group were also handled every day and put on a stopped treadmill. Body weight was measured weekly during the training period. Running capacity tests were performed on each rat at the beginning of the protocol and on week 6, for adjustment of exercise intensity and assessment of increase in performance.

### Euthanasia and sample collection

At the end of the training period, the animals were sacrificed by inhalation of CO_2_ and exsanguination by puncture of the vena cava. Blood samples were collected in heparinized syringe and centrifuged (3500 rpm/10 min/4°C) to obtain the plasma, which was stored at -80°C. Later, the aortas were removed and immediately immersed in cold Krebs-Henseleit solution, and the hearts were weighed.

### Thiobarbituric Acid Reactive Substances (TBARS)

TBARS levels were measured according to a method adapted from Yagi.^21^ Briefly, the lipid peroxidation was determined by the reaction of malondialdehyde (MDA) with thiobarbituric acid (TBA) to form a pink chromogen that can be quantified by spectrophotometry (in 532 nm). The values of absorbance detected in the samples were interpolated to a tetramethoxypropane standard curve (0 to 100 *µ*M).

### Plasma antioxidant capacity (Ferric Reducing Ability of Plasma FRAP)

The method described by Benzie & Strain^22^ is based on the ability of plasma to reduce Fe^+++^ to Fe^++^ ions in the presence of 2,4,6 tripyridyl-s-triazine (TPTZ) at low pH with the formation of Fe^++^-tripyridyltriazin, a blue colored complex. Before the beginning of the experiments, three solutions were prepared: A (Acetate buffer: 300 mM, pH 3.6 and 40 mMHCl), B (TPTZ - 2,4,6-tri- [2-pyridyl]-s-triazine - 10 mm) and C (FeCl_3_.6H_2_O - 20 mM). The working reagent was prepared by adding A + B + C in the ratio 10: 1: 1 (V/V). Later, the plasma samples (0,08 mL) were added to the mixture of deionized water (2,4 mL) and working reagent (0.25 ml). This solution was placed in microplates in parallel with the blank sample (only working reagent) and the standard curve samples (FeSO_4_ 0-1000 mmol/L). These samples were read in spectrophotometer at 593 nm, and the concentrations (in uM/L) were calculated by interpolation in the standard curve.

### Organ bath studies

In a Petri dish covered with paraffin containing Krebs-Henseleit solution, the aortas were carefully divided into rings (3-5 mm). These rings were, then, set in 2 mL organ baths, fixed to a lower stainless steel hook attached to a stationary support and to an upper one connected to an isometric force transducer. The organ bath contained Krebs-Henseleit solution of the following composition (mM): NaCl 130; KCl 4.7; CaCl_2_ 1.6; KH_2_PO_4_ 1.2; MgSO_4_ 1.2; NaHCO_3_ 15 and glucose 11.1. The Kreb-Henseleit solution was kept at 37ºC, pH 7.4 and continuously bubbled with a mixture of 95% CO_2_ and 5% O_2_. Tension was continuously monitored and recorded using a Powerlab 8/30 data acquisition system (Australia ADInstruments). Prior to the addition of drugs, the rings were equilibrated for 60 minutes under a resting tension of 1.5 g.

All preparations were challenged with 10^-4^ mol/L acetylcholine (ACh), after precontraction induced by10^-5^ mol/L phenylephrine (Phe), to verify the endothelial integrity. Some preparations had their endothelium mechanically removed, which was confirmed by the absence of relaxation in response to ACh. Later, both intact and endothelium-denuded preparations were challenged with cumulative concentrations of norepinephrine (NE; 10^-10^ - 10^-4^ mol/L). Intact preparations were also challenged with cumulative concentrations of NE in presence of N_ω_-Nitro-L-arginine methyl ester hydrochloride (L-NAME) 10^-4^ mol/L, a non-selective NOS inhibitor added 20 minutes before the challenging. In parallel, intact preparations were challenged with single concentrations of ACh (10^-4^ mol/L) after precontraction induced by10^-5^ mol/L Phe.

The evoked responses (in g) to the aforementioned vasoactive agents, cumulatively added into the organ bath, were plotted to obtain concentration-response curves. Non-linear regressions (variable slope) of these curves revealed the R_max_ (maximal response; highest point of each concentration-response curve) and the pEC_50_ (negative logarithm of the concentration that evoked 50% of the maximal response). The pEC_50_ is indicative of the sensitivity to the studied drug.

The following drugs were used: Acetylcholinechloride;L-(-)-norepinephrine(+)-bitartrate salt monohydrate, N_ω_-Nitro-L-arginine methyl ester hydrochloride, and phenylephrine hydrochloride, all purchased from Sigma Chemical Co.

### Statistical analysis

Data are reported as mean ± standard error of the mean (SEM). Data obtained from the CEx and AEx groups were compared independently with those obtained from SED group by unpaired Student "t" test. Before applying the Student "t" test, the Gaussian distribution of data was verified by the Shapiro-Wilk normality test. The statistical analysis was performed using the GraphPad Prism 6.0 software. P values less than 0.05 were considered statistically significant.

## Results

### Running capacity test

**Figure 1:**
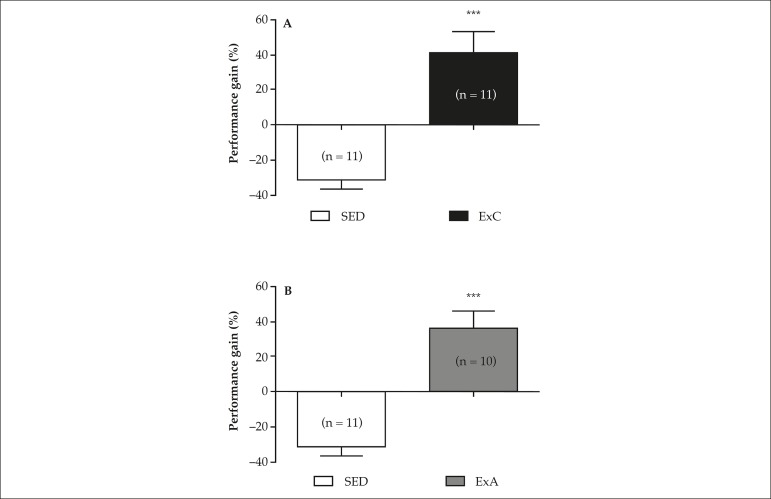
Performance gain (%) of animals submitted to continuous exercise training (CEx; A) or accumulated exercise (AEx; B) in comparison to sedentary animals (SED). Columns represent mean ± SEM; in parenthesis, number of independent determinations. *** p < 0.001 compared to SED animals (unpaired Student's t-test).

During the training period, a significant improvement in running performance was observed in the CEx and AEx groups (Figure 1A and 1B) as compared with the SED animals. In contrast, in the same period, a reduction in performance was observed in the SED group.

### Body and heart weight

During the training period, body weight gain was significantly (p < 0.05) lower in CEx animals (11.69 ± 3.28%; n = 11) than in SED animals (21.38 ± 1.19%; n = 11). On the other hand, body weight gain in AEx animals (21.38 ± 1.19%; n = 11) was not statistically different in comparison with SED animals. Heart weight in CEx (1.30 ± 0.04 g; n = 11) or AEx (1.37 ± 0.05 g; n = 11) animals was not significantly different from that in SED animals (1.38 ± 0.05g; n = 11).

### TBARS and FRAP

The TBARS values in CEx and AEx animals [17.85 ± 3.57 (n = 11) and 24.91 ± 5.18 (n = 11), respectively] were not significantly different in comparison with SED animals [20.88 ± 5.29 (n = 11)].

Both continuous and accumulated exercise had no effect on plasma antioxidant capacity, since FRAP values in CEx and AEx groups [1309.00 ± 74.04 (n = 11) and 1222.00 ± 55.98 (n = 11), respectively] were not significantly different than in SED animals [1215.00 ± 57.11 (n = 11)].

### Vascular responses

**Figure 2:**
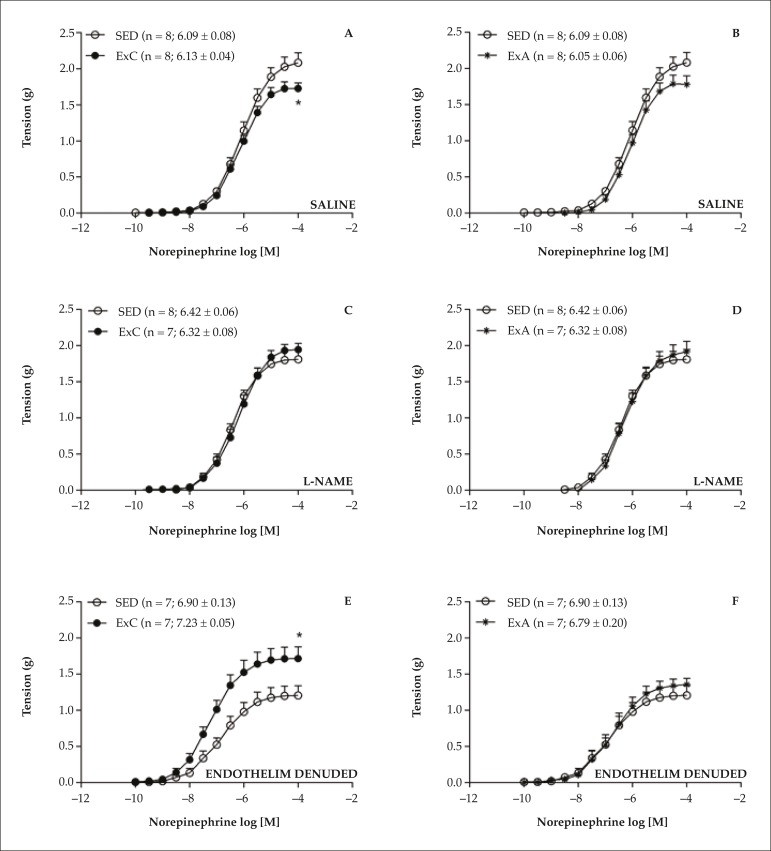
Concentration-response curves to norepinephrine determined in intact thoracic aorta preparations obtained from animals trained by continuous (ExA) or accumulated exercise (ExA), in comparison to sedentary animals (SED), not treated (A and B) or treated with 10^-4^ mol/L L-NAME (C and D) as well as in not treated endothelium denuded thoracic aorta preparations (E and F). In parentheses, the number of independent determinations (n) followed by pEC50 values. Data in mean ± SEM. * p < 0.05 compared with the SED group ("t" test of Student).

The CEx reduced the magnitude of responses to NE in the aorta, with a significant reduction in R_max_ values compared to SED animals. However, no significant differences in pEC_50_ were observed between these groups (Figure 2A). This reduction in NE R_max_ was not observed in L-NAME pre-treated preparations (Figure 2C) or in preparations without endothelium (Figure 2E). On the other hand, the slight reduction of NE responses induced by ExA did not result in significant reduction of R_max_ or pEC_50_ (Figure 2B), and was suppressed by the presence of L-NAME (Figures 2C and D) or the endothelium removal (Figures 2E and F).

**Figure 3:**
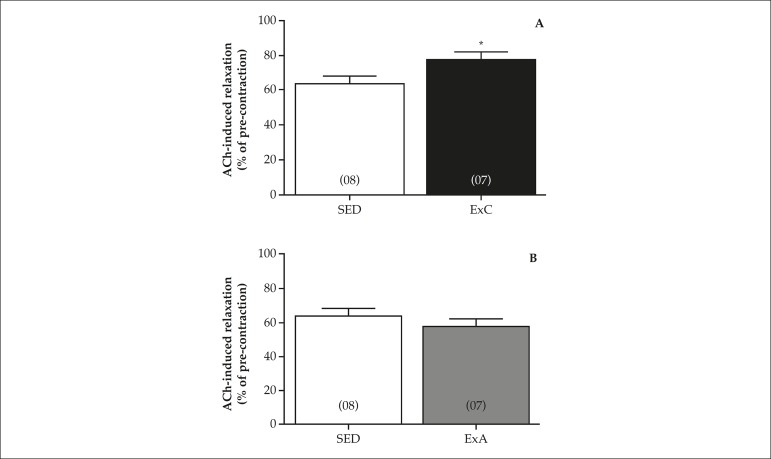
Relaxation induced by acetylcholine (ACh; 10^-4^ mol/L), in % of phenylephrine-induced pre-contraction (Phe; 10^-5^ mol/L), in animals submitted to continuous exercise (ExC; A) or accumulated exercise (ExA; B) training, in comparison with sedentary animals (SED). Columns represent mean ± SEM; in parenthesis, number of independent determinations. *p < 0.05 compared to the SED animals (unpaired Student's t-test).

Moreover, the CEx also increased the 10^-4^ mol/L ACh-induced relaxation of intact aorta precontracted with 10^-5^ mol/L Phe (Figure 3A). This effect was not observed in AEx (Figure 3B).

## Discussion

The practice of regular exercises has been proven effective in reducing the risk of cardiovascular diseases.^3^ However, the concept that only intense and long-lasting sessions of exercises are beneficial to health may compromise the adherence to this practice.^23^Indeed, the flexibility of exercise plan, including intensity, duration and frequency, can lead to improved adherence.^6,24^ The practice of short, but repeated exercise bouts throughout the day, may be an alternative way to get exercise benefits.^3^

Regarding cardiovascular diseases, further studies are needed to confirm the beneficial effects of exercise accumulated in several short bouts on vascular endothelium. In this context, we compared one continuous bout of exercise (1 hour/day) with the same amount of exercise distributed in short, repeated bouts (4 bouts/day), to assess the beneficial, cumulative effects of exercise. Although it was not the objective of this study to propose an exercise program that could be used in humans, which limits the extrapolation of our results to humans, the study raises the discussion about the usefulness of accumulated exercise in the clinical practice.

Interestingly, in the present study, not only continuous exercise, but also accumulated exercise increased the animals' running capacity on the treadmill. Despite the limitations of experimental models to reproduce exercise training protocols designed for humans, these findings suggest that the positive effects of accumulated exercise on fitness observed in animals, may also occur in humans.^11^ For example, accumulated exercise can be an alternative approach to help individuals to get away from a sedentary lifestyle.

The improvements in running capacity on treadmill were not accompanied by changes in heart weight or in TBARS or FRAP values. These results indicate that, although the CEx and the AEx protocols were not able to increase the antioxidant defenses, no significant changes in plasma levels of free radicals were observed either. This may be explained by the intensity of exercise applied - 50-60% of maximum capacity - which is considered moderate.

In addition, accumulated aerobic exercise has been suggested as a strategy in weight control.^6,13^It has been proven effective in reducing blood pressure,^7,12^post prandial triglycerides,^10^ skinfold thickness and waist circumference,^6^ and in increasing high-density lipoprotein levels.^8,9^ In the present study, however, as compared with SED group animals, body weight gain was significantly lower in the CEx group but not in the AEx group.

Exercise may also increase the laminar blood flow on endothelial surface, thereby increasing the shear stress on this surface.^25^An increased shear stress on the endothelium may induce the expression of several enzymes involved in the synthesis of substances that regulate vascular tone, local oxidative balance, coagulation process and endothelial inflammation.^26,27^Therefore, exercise may increase the endothelial production of vasodilator substances, and hence modulate the NE responses in several vascular beds, including rat aorta.^14,16^

The present study showed that the CEx decreased the responses to NE in aorta with a reduction of R_max_, indicating an improvement in endothelial function. The main endothelium-derived relaxing factor is NO, a diffusible gas synthesized mainly by eNOS in the vascular endothelium. In our study, the reduction of R_max_ was suppressed by L-NAME, a non-selective NOS inhibitor, or reversed in preparations without endothelium. Since the eNOS expression may be increased by shear stress, thereby increasing the synthesis of NO,^18,20,27^ the reduction of NE responses in rat aorta induced by the CEx may be due to a greater efficiency of endothelial NO-related mechanisms, resulting from an increased expression of endothelial eNOS. The involvement of endothelium-derived NO in the reduction of NE responses in aorta of animals exposed to CEx has been also described by other studies.^16,20,28^

The increased NE response, characterized by elevation of R_max_ and pEC_50_, induced by CEx in aorta preparations without endothelium was unexpected, but also reinforces the pivotal role of the endothelium in the modulation of this response in these preparations. In addition, it has been proposed that NO is the principal mediator of the ACh-induced relaxation in rat aorta preparations.^29^ In this manner, as previously described, the increased ACh-induced relaxation corroborates the involvement of NO-related mechanisms in the endothelium in these preparations.

Unfortunately, evidence of an exercise-induced enhancement in endothelial function has been demonstrated only by continuous or intermittent exercise studies. Evidences of a direct effect of AEx on the endothelium are scarce in the literature. Thus, once verified that CEx may improve the endothelial function in our experimental conditions, we began to investigate whether splitting the exercise in short bouts (with a total time corresponding to one continuous session) promoted similar effects on the endothelium. No significant increases in NE and ACh responses, induced by AEx were found in rat aorta preparations, suggesting that this type of exercise has no effect on endothelial function. It is worth mentioning that previous reports have shown that the effects of shear stress on the enzymes involved in the endothelial production of modulatory substances depends on time of exposure to exercise.^18,19,27^ Our results suggest that the beneficial effects of exercise on endothelial function are achieved only if exercise are practiced for a long enough period.^30,31^ However, the minimum time required for such effects remains to be determined.

The findings on the effect of AEx on vascular endothelium are not conclusive and may not be extrapolated to the clinical setting, since, to our knowledge, this is the first study investigating a direct effect of AEx on vascular endothelium in experimental conditions. Yet, in the present study, the concentration-response curves to NE had a descending trend. Thus, the improvement in endothelial function induced by AEx may occur in animals with endothelial dysfunction caused by aging, hypertension, atherosclerosis or diabetes and those chronically exposed to alcohol and/or smoke.^32^ In fact, one of the few studies on the direct effect of AEx on the endothelial function was performed in adolescent boys submitted to an ingestion of a high-fat breakfast and lunch. This diet has induced endothelial dysfunction in these boys, which was reversed by short bouts of exercise that were repeated throughout a day.^33^Also, it was demonstrated that 30 min walk divided into sessions of 10 min (with intervals of 50 minutes to rest) was effective in reducing systolic blood pressure in pre-hypertensive individuals.^34^ Such reduction in blood pressure may be involved at least partially in the exercised-induced improvement of endothelial function. Thus, further studies on endothelial dysfunction models are required to better understand the therapeutic potential of AEx.

## Conclusion

In conclusion, the continuous and accumulated exercise protocols employed in this study increased the fitness of the animals, which suggests the usefulness of the AEx as a strategy to introduce people to physical training programs. However, as compared with CEx, AEx was not as effective in preventing body weight gain or improving the endothelial function of aorta of these animals.
